# Phylogenetic Detection of Recombination with a Bayesian Prior on the Distance between Trees

**DOI:** 10.1371/journal.pone.0002651

**Published:** 2008-07-09

**Authors:** Leonardo de Oliveira Martins, Élcio Leal, Hirohisa Kishino

**Affiliations:** 1 Graduate School of Agriculture and Life Sciences, University of Tokyo, Tokyo, Japan; 2 Federal University of São Paulo, São Paulo, Brazil; Center for Genomic Regulation, Spain

## Abstract

Genomic regions participating in recombination events may support distinct topologies, and phylogenetic analyses should incorporate this heterogeneity. Existing phylogenetic methods for recombination detection are challenged by the enormous number of possible topologies, even for a moderate number of taxa. If, however, the detection analysis is conducted independently between each putative recombinant sequence and a set of reference parentals, potential recombinations between the recombinants are neglected. In this context, a recombination hotspot can be inferred in phylogenetic analyses if we observe several consecutive breakpoints. We developed a distance measure between unrooted topologies that closely resembles the number of recombinations. By introducing a prior distribution on these recombination distances, a Bayesian hierarchical model was devised to detect phylogenetic inconsistencies occurring due to recombinations. This model relaxes the assumption of known parental sequences, still common in HIV analysis, allowing the entire dataset to be analyzed at once. On simulated datasets with up to 16 taxa, our method correctly detected recombination breakpoints and the number of recombination events for each breakpoint. The procedure is robust to rate and transition∶transversion heterogeneities for simulations with and without recombination. This recombination distance is related to recombination hotspots. Applying this procedure to a genomic HIV-1 dataset, we found evidence for hotspots and de novo recombination.

## Introduction

A variety of distinct methods have been developed to detect recombination (for a review see [1]). They can be broadly classified into two classes, depending on the relative contributions of the recombinational and mutational processes [2]: the population genetic approach and the phylogenetic approach [3]. The population genetic approach uses the information of the linkage disequilibrium among segregating sites, assuming ubiquitous recombination. The linkage disequilibrium depends not only on the recombination rate between the sites but also on the the population history. Recombination rate and the population history are then estimated by introducing the ancestral recombination graphs (ARGs) as nuisance parameters (i.e., the population histories are averaged over all possible particular recombination scenarios) [4]–[12]. The population genetic approach is efficient when recombination is pervasive along the genome, disrupting the phylogenetic signal. In this context recombination hotspots can be detected as regions where the recombination rate is higher than the local background rate [4], [13].

When the recombination rate is moderate compared with mutation rate, the sequences may be decomposed into a few segments that have specific phylogenetic histories. Instead of treating the recombination history as a nuisance parameter, the phylogenetic approach estimates the breakpoints and the phylogeny of the segments, assuming that some phylogenetic structure is preserved. Many techniques are based on sliding window procedures that compare the topology of one segment against neighboring segments or the whole alignment. This comparison may be based on the phenetic distance [14]–[16], likelihood [17] or posterior distribution [18] of the topologies for each arbitrary segment. Hidden Markov Models [19], [20] regard topologies at sites as hidden states, where the transition probability penalizes the inconsistency of topology between neighboring sites. Bayesian change point models [21], [22] identify recombination breakpoints and differentiated substitution rates as change points of topologies and evolutionary rate parameters. While these Bayesian procedures have a sound statistical background, they can not reliably estimate the history of recombination events when the number of taxa increase, due to the large degree of freedom on topologies.

Here, we present a new method to detect recombination based on the disagreement of topologies from adjacent segments of DNA alignments. Our approach falls into the category of phylogenetic approaches, and we consider only recombinations that influence the topology. We conceived an algorithm that approximates the minimum number of subtree prune-and-regraft (SPR) operations required to resolve inconsistencies between two competing unrooted trees. This number is called the SPR distance (*d_SPR_*). We refer to our algorithm as the (approximate) SPR distance (*d̂* *_SPR_*). The distributions of distances between adjacent segments are then used as a prior in a Bayesian approach to penalize highly discordant topologies between two neighboring segments. Consequently, this approach reduces the topology space explored for each segment, thereby reducing the computational burden. Additionally, because inconsistent topologies are constrained by the distances of neighboring segments, the uncertainty in the estimation is largely reduced. It is possible to extract well-resolved trees even from short non-recombinant DNA regions within an alignment. Since the distances can be obtained from unrooted trees, our method does not assume a known outgroup. The posterior distribution of distances and the topologies of the segments make it possible to interpret the recombination history. Therefore, our procedure may work well for the exploratory analysis of identifying recombination patterns.

We evaluated our method initially by analyzing sets of simulated alignments in the presence and absence of recombination. The results suggest that this is a reliable method to detect and distinguish recombination from rate heterogeneity in simulated data. We then used our method to study recombination in empirical sequences from HIV-1. Recombinant HIV-1 variants that spread epidemically throughout a population of unrelated individuals are designated circulating recombinant forms (CRF), and genomes of CRF viruses are mosaics comprised by regions derived from two or more distinct parental subtypes. These recombinants are routinely detected by phylogenetic methods based on a local similarity between the putative recombinant and all possible parental sequences [14], [15]. Although in South American countries subtype B remains the most prevalent clade of the HIV-1 infection, there are great varieties of different BF recombinants (as a result of recombination between subtypes B and F) co-circulating in these countries [23]. In this context, it is expected that recombinations among HIV-1 BF variants will occur frequently and that these events are currently neglected by methods that exploit the mosaic pattern based on sequence parentage. For this reason, we explored the pattern of recombination in BF viruses from South American countries in more detail. Our method provides evidence that the extent of recombination in HIV-1 can be underestimated if one relies solely in the mosaic pattern dictated by the reference parentals.

## Results

### Approximate SPR distance between topologies

**Figure 1:**
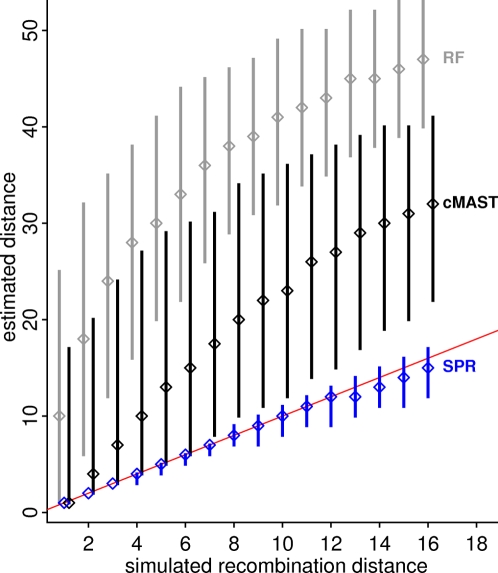
Comparison of tree distance metrics for topology pairs over 64 taxa. The vertical bars represent the 95% range, and diamonds intercept the median over 5000 replicates for each simulated distance. The cMAST estimates (black) were calculated with PAUP [33], while the Robinson-Foulds (RF) distance (gray) and our *d̂_SPR_* approximation (blue) were computed using in-house software. The diagonal line (red) represents the case where the estimate and true values agree. On the horizontal axis, we have the “real” number of SPR moves applied (ranging from one to 16) on random topologies, and, on the vertical axis, we show the estimated distances using the RF, cMAST and *d̂_SPR_* methods.

To evaluate the performance of our approximate SPR distance (*d̂_SPR_*) algorithm, we applied subtree prune-and-regraft (*SPR*) moves on a random topology and then estimated the distance between the original and rearranged topologies [24]. Figure 1 shows the distribution of estimated distances obtained by the complement Maximum Agreement Subtree (cMAST, number of leaves causing the disagreement) by the Robinson-Foulds (number of edges in disagreement) method and by our *d̂_SPR_* method for topologies with 64 taxa (the largest number our implementation can handle). We performed 1–16 SPR moves (“real” *d_SPR_*) in this analysis, with 5000 replicates for each distance. The approximation *d̂_SPR_* is very good for small values of *d_SPR_*, and we observed a lower performance when the “real” number of SPR moves increases. The same behavior was observed for smaller trees, with the observation that performance decreased faster in this case (results not shown). Conversely, estimates obtained by cMAST or Robinson-Foulds non-linearly overestimate the number of SPRs in most cases. The procedure always gave the correct answer for simulations of one SPR, which means that our procedure is conservative since it does not report a distance larger than one (several moves) if the topologies can be explained by one SPR event. The sub-optimal performance is the result not only of the heuristic nature of the algorithm but also of the inability in simulating topologies with an exact SPR distance [25]. The calculation of *d̂_SPR_* for this analysis (8×10^4^ simulated tree pairs) took 100 seconds on a Pentium M 1.6GHz running Debian GNU/Linux. A panel with individual histograms for this comparison can be seen in Figure S1.

By applying several SPRs on a topology, it does not guarantee that the final topology can be explained by fewer than the number of applied moves [25], [26]. In our simulations, we tried to circumvent this problem by allowing branches to participate in only one SPR move and by simulating recombination on large phylogenies. Other strategies based on exploiting the SPR neighborhood of topologies [26] gave similar results with a much higher computational burden for simulation (results not shown).

### Recombination detection on simulated sequences

To evaluate the performance of our method in detecting recombination, we simulated datasets with eight and 12 taxa while mimicking DNA sequences with recombination breakpoints. To do this, we simulated fragments of sequences assuming a defined evolutionary model (tree and parameters) using PAML [27]. We used the HKY model (π_A_ = 0.3, π_G_ = 0.4, π_C_ = 0.2, π_T_ = 0.1), where each branch length was drawn from a uniform distribution between 0.2 and 1 and then rescaled. We simulated 100 replicates under the same evolutionary model for each scenario. Each fragment was simulated independently and then concatenated into a single alignment. As a result, the simulated alignments corresponded to mosaic DNA sequences formed by distinct non-recombinant fragments. Each fragment included in the alignments was simulated from distinct topologies (distinct evolutionary history). Therefore, the concatenation mimics the effect of natural recombination. Consequently, the simulation approach resembles natural recombination that occurs in organisms that exchange large genomic regions between distinct lineages. In addition, the heterogeneity of branch lengths resembles a relaxed molecular clock process, simulating heterogeneity among lineages.

**Figure 2:**
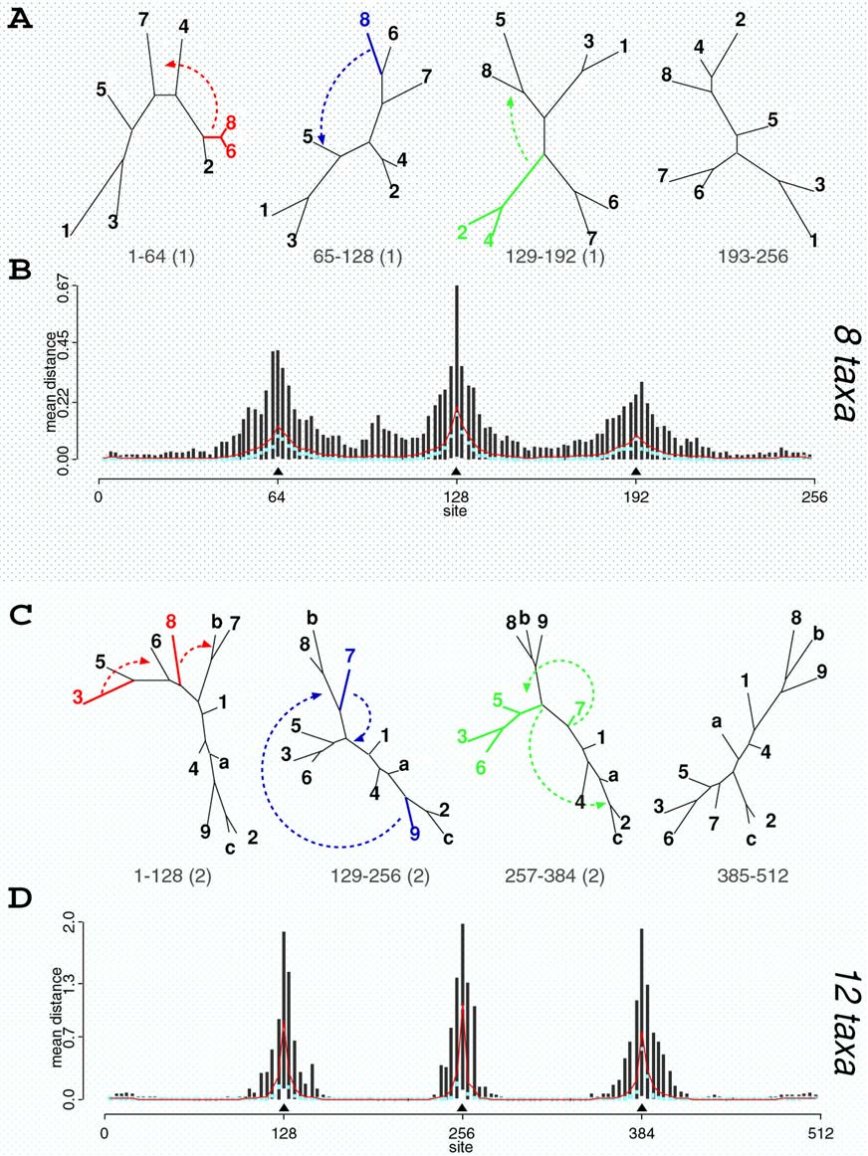
Bayesian analysis on simulations of eight (panels A and B) and 12 (panels C and D) taxa. Panels A and C show trees used in the simulations. Branch lengths are proportional to the amount of evolution between nodes. Numbers below trees show site ranges over which the topologies were used, with true *d_SPR_* to the next topology in parenthesis. Disagreements between segments can be explained by one SPR between trees for the eight taxa scenario and two SPR moves between 12 taxa trees. From left to right, one possible SPR explanation is represented by arrows. The distributions of posterior mean SPR distances per segment over 100 simulated datasets (for each scenario) are shown in panels B and D. The black vertical lines are the 95% inter-quantile ranges, while the light blue dots are the median values over all datasets. The red lines are the mean values of the average SPR distance per segment. The true recombination breakpoints are represented by filled triangles.

Figure 2A shows the simulation strategy for eight sequences, where each non-recombinant fragment is composed of 64 base pairs (bp). The concatenated alignment then has 256 bp with three known breakpoints. For this simulation, we fixed kappa (*κ*) to 1.4 and rescaled branch lengths such that each site had, on average, one substitution. This apparent high value reflects our assumption that some phylogenetic signal is present, and the non-recombinant fragments are short. MCMC analysis was conducted with 5×10^4^ iterations (after 5×10^3^ iterations were initially discarded), with 100 samples from the posterior being drawn. For this analysis, we assumed that each segment was composed of 2 bp such that we sampled from 128 segments.

For 12 taxa simulation, each non-recombinant region (128 bp) supports not only a different topology but also distinct evolutionary parameters. The average rate per site of each 32 bp region was scaled to be between one and four, and *κ* was set to a random number between one and two. In such a scenario, our simulations take into account rate heterogeneity among sites and lineages. Likewise, we simulated non-recombinant fragments of 128 bp following the topologies displayed in Figure 2C, and the fragments were then concatenated into an alignment of 512 bp. We noticed that, at each recombination breakpoint, at least two recombinations (SPR moves) were necessary to explain differences between non-recombinant regions. In this case, the MCMC analysis was conducted with 2×10^4^ iterations (sampling at each 200 iterations), and we assumed each segment to be composed of 4 bp, containing 128 segments in total.

If, for each replicate, we look at the average posterior SPR distance per segment (actually, the distance between each segment and the next), we will have the distribution of the mean distances for each segment for 100 replicates. The distribution of average posterior distances for the simulation with eight and 12 taxa are illustrated in Figure 2 (panels B and D, respectively). Considering the regions surrounding the true breakpoints (filled triangles), we observe that breakpoints, as estimated by a mean distance larger than zero, are usually found within 20 bp from the true ones. If we sum the mean values around the peaks (red lines), we can find the true number of SPR moves between the regions. In fact, for each posterior sample we can sum up the individual distance values (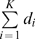) to obtain the total number of SPRs (lower bound for the minimum number of recombinations) and the count of how many segments have *d̂_SPR_* larger than zero (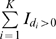, where *I_x_* is the indicator function) that will give us the number of recombination breakpoints. For each dataset, we have the distribution of the number of SPRs and breakpoints. The mode values inferred the true number of SPR operations in 71% of the datasets and correctly predicted the number of breakpoints in 84% of the simulations with eight taxa. For simulations with 12 taxa, it was successful in detecting a total number of six SPRs in 80% of the replicates and a total number of three breakpoints in 94% of the datasets (data not shown). By summing up the fraction of posterior samples where *d̂_SPR_* is larger than zero over a region we have the posterior probability of a breakpoint over this region. By repeating this procedure for all replicate datasets over a 20 bp region around the true breakpoints, we found the estimated breakpoint locations to be within 20 bp of the true values on 63% of the eight taxa datasets and on 91% of the simulations with 12 taxa, on average. The individual values for the first, second and third breakpoints are respectively 62%, 76% and 51% for eight taxa and 91%, 96% and 86% for 12 taxa. Each dataset on eight taxa took, on average, less than eight minutes to analyze, while each of the 12 taxa simulations took approximately 15 minutes to complete on a Pentium M 1.6 GHz running Debian GNU/Linux.

### Inferred versus true trees

**Figure 3:**
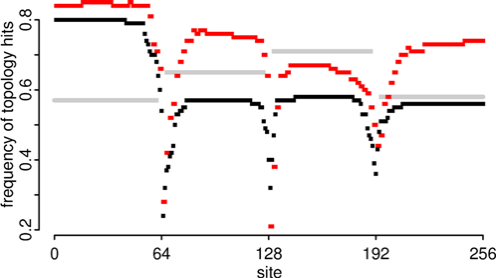
Accuracy of Bayesian methods in reconstructing true topologies. The red dots represent the fraction of simulated datasets for each segment, where the MAP topology estimated using our method corresponds to the true topology over 100 datasets. The black dots represent the accuracy of the cBrother software in obtaining the true topologies based on the MAP structure. The gray horizontal bars represent the same quantity for the independent analyses of each non-recombinant region (of 64 bp) using the software MrBayes (over 100 datasets).

To check the frequency at which the true topology was reconstructed, we compared, for each dataset, the maximum *a posteriori* (MAP) topology of each segment against their respective true trees (i.e., the trees initially used to simulate the datasets). From this comparison, we counted the number of times the topologies agreed in all datasets, which gave us the frequency of topology hits per segment. The results for eight taxa datasets are shown in Figure 3. In this figure, we show the same statistic (proportion of correctly reconstructed topology) using cBrother software [28], a fast implementation of the Bayesian procedure DualBrothers [21]. DualBrothers (and cBrother) is a Bayesian phylogenetic procedure for recombination detection [29] and cBrother is capable of relaxing the parental assumption [28] accurately working with up to six or seven taxa [30]. The output from cBrother analyzed was the MAP number of breakpoints and mosaic structure, namely the most frequent combination of topologies and breakpoints. Here, we report that cBrother performed well with eight taxa, finding the true topology in 60% of the simulations-median over sites (Figure 3). Conversely, our procedure outperformed cBrother, given that, for most sites, the MAP topology corresponded to the true topology in 73% of the simulations (red dots in Figure 3). For datasets with 12 taxa, the MAP topologies using our procedure reconstructed the true ones in 75% of the segments, on average (data not shown). If we consider only the detection of recombination, cBrother retrieved the correct number of breakpoints in 55% of the datasets with eight taxa, much lower than the 84% reported by our procedure (not shown).

Both methods have decreased performance around the recombination breakpoints, where the phylogenetic signal is conflicting. The superior performance of our method is due to the penalty against distant topologies since cBrother needs to consider equally all (2*n*−5)!! topologies over *n* taxa without parental assumptions. We also compared the results described above with MrBayes, a Bayesian procedure used to infer the posterior distribution of topologies in the absence of recombination [31], [32]. For each simulated dataset, we used MrBayes to infer the posterior distribution of topologies for each of the four 64 bp non-recombinant fragments independently. The results, depicted in gray in Figure 3, show that MrBayes is less effective than our method in recovering the true tree, except for the region between sites 129 and 192. Our procedure does not assume that breakpoints are known, but outperformed MrBayes applied to each fragment free from recombination. This shows that MrBayes, similar to cBrother (but to a lesser extent), suffers from the large topology space and that the prior on the SPR distance is effective to reduce the degree of freedom. Another approach (but difficult to implement with recombinant datasets) would be to analyze consensus topologies instead of MAP topologies. We also inferred the topologies of the non-recombinant fragments using minimum evolution and maximum likelihood criteria [33], reconstructing the true trees respectively in 53% and 60% of the simulations, on average (results not shown).

### Rate heterogeneity in absence of recombination

**Figure 4:**
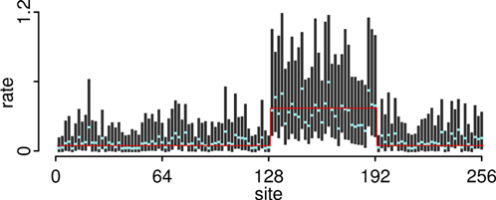
Bayesian analysis of one simulated dataset with single topology and model heterogeneity. The panel shows the distribution of average substitution rates *μ_i_* per branch for each segment. Dark gray vertical bars represent the 95% credibility interval, and light blue points represent the median values. The true values are depicted by red horizontal lines.

Even in the absence of recombination, spatially structured model heterogeneity can lead to falsely detected recombination [21]. To evaluate the robustness of our method against the bias induced by rate heterogeneity, we simulated a 256 bp alignment with eight taxa where all sites share the same topology (first topology in Figure 2A). Furthermore, the average substitution rate per site was set to 0.6 for almost all segments, with the exception of sites 129 to 192, where the average rate was fixed at 4.8 substitutions per site; *κ* was set to 1.4 for all sites (Figure 4). No evidence of recombination was found in any of the 100 datasets (results not shown). Since our procedure integrates out individual substitution rates over branches, the parameter *μ_i_* is the average substitution rate per branch. Thus, an average of 0.6 substitutions per site over eight taxa implies that *μ_i_* = 0.046 since we have 2×8−3 = 13 branches. Our primary interest, however, is not the estimation of individual site rates, but our method seems robust enough to model heterogeneity. In our model, the independence of rates between segments accommodates this heterogeneity while avoiding over-parameterization caused by individual branches.

### Simulation of a recombination hotspot

**Figure 5:**
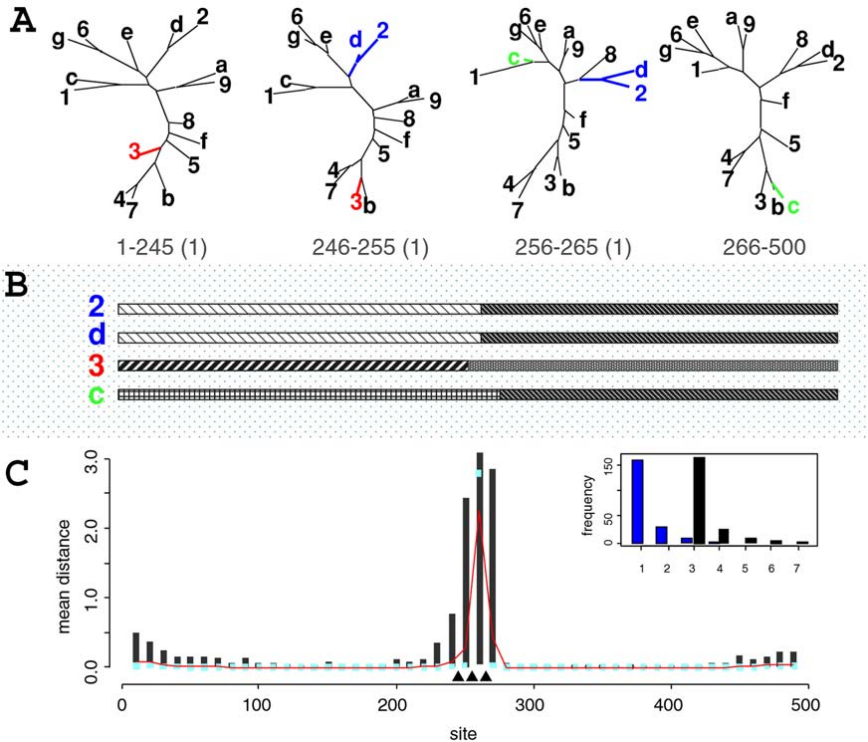
Simulation of a hotspot region. Panel A shows the topologies used in the simulation, where the number ranges represent the site regions, and the numbers in parenthesis represent the SPR distance to the next tree. One possible recombination scenario is shown with colored subtrees. Panel B shows the mosaic structure of the four recombinant sequences, highlighting the nearness of the breakpoints. Panel C shows the distribution of average SPR distances assuming 10 bp segments, with median (light blue dots) and 95% inter-quantile ranges (dark gray bars) (over 200 datasets). The filled triangles represent the true recombination breakpoints. On the inset of panel C, the histogram of the total number of SPR distances (in black) and the number of breakpoints (in blue) based on the modal values over 200 datasets is shown.

The previous simulations with 12 taxa provide a scenario in which each breakpoint can be considered a hotspot because at least two SPRs are necessary to explain the inconsistency between neighboring regions and because the true number of recombinations will always be larger than the unrooted *d_SPR_*. We are also interested in the scenario in which a hotspot can be represented as unusually clustered adjacent breakpoints since we suspect that distinguishing both might be difficult given the stochastic error on the breakpoint locations. Therefore, we simulated datasets with 16 taxa having three recombination breakpoints at a distance of 10 bp between each other in a 500 bp alignment. The SPR distance between adjacent trees is one, giving a total of three SPR events. This scenario is represented in Figure 5A, where one possibility is that the recombinant sequences are those labeled 2, 3, *c* and *d*. Notice that it is of equal likeliness that sequence 3 is a parental and sequence *b* (or sequences 4 and 7) is the recombinant. The mosaic structure for these sequences is depicted in panel B. For example, the sequence *c* is a recombinant between sequences 1 and *b*, and the recombination breakpoint is between sites 265 and 266. In the same way, the ancestor of sequences 6, *e* and *g* recombined with the ancestor of taxa eight between sites 255 and 256, resulting in the extant sequences 2 and *d*. Note that, in this case, there are two recombinant sequences sharing one ancestral recombination.

We therefore simulated 200 replicate alignments under this scenario with an average number of substitutions per site randomly sampled between two and five (rate heterogeneity) and a transition∶transversion ratio between one and four for each 5 bp region. In the Bayesian analyses, we assumed segments of 10 bp, such that the true breakpoints would lie within segments. This is likely to happen in practice, mainly in large segments where the true breakpoints will not lie in the segment border. The summary of the analyses is shown in Figure 5C, in which the average SPR distances are larger than zero only in the region under recombination. Since breakpoints are clustered, our method could not locate their exact location in the alignment. We also observed that the procedure correctly detects the total number of SPR events, corroborating the hypothesis of a recombination hotspot. This lack of resolution in pinpointing the breakpoint locations is not an artifact of the misplaced segment locations since a similar behavior is observed for 5 bp segments (data not shown). It is, in fact, the result of the lack of phylogenetic signal since there are only 10 bp supporting the intermediate topologies. In our procedure, as long as there is some phylogenetic structure (in this case, in the border regions), it is possible to quantify the number of recombinations even when the breakpoints cannot be precisely located. The true first and last topologies were found as the MAP topologies in 82% and 85% of the datasets, respectively.

### HIV-1 recombination in South America

To validate our procedure with experimental DNA sequences we analyzed near full-length HIV-1 genomes. We first selected BF recombinant sequences with similar mosaic patterns. These sequences were selected from an alignment of South American BF recombinant sequences comprising 8402 bp. We compared each one independently against reference subtypes F, B and C using the DualBrothers software. By repeating the DualBrothers analysis for each putative recombinant, we assume that the parental sequences are not involved in recombination, an unnecessary feature in our method. Our final dataset consisted of eight BF recombinant sequences with similar mosaic patterns plus three reference subtype sequences, which were then analyzed at once using our procedure. The recombinant sequences represent the reference circulating recombinant form CRF12_BF (according to the Los Alamos HIV databank) and are described in the Supplemental Table S1.

We ran the sampler for 10^4^ iterations as a warm-up and then ran 5×10^5^ iterations sampling 1000 times on the 11 sequences alignment. The initial states for the warm-up were chosen based on five cycles of 500 iterations of simulated annealing with final temperature of 1.2 (initial temperature of 0.2). In this analysis, we assumed 10 bp segments, and, since the genomic alignments are composed of 8402 bp, we have 840 segments. This procedure was repeated for two independent chains to access convergence from overdispersed starting points. The results reported here are based on the pooled chains. The starting point (initial state) was, in fact, the same, but the simulated annealing stage disperses these states. The convergence was accessed by visual inspection of the time series of the samples, posterior distribution and the scaled reduction factor [34] for the posterior probability, the number of breakpoints and the total estimated number of SPR moves. Each run took approximately 24 hours to complete.

**Figure 6:**
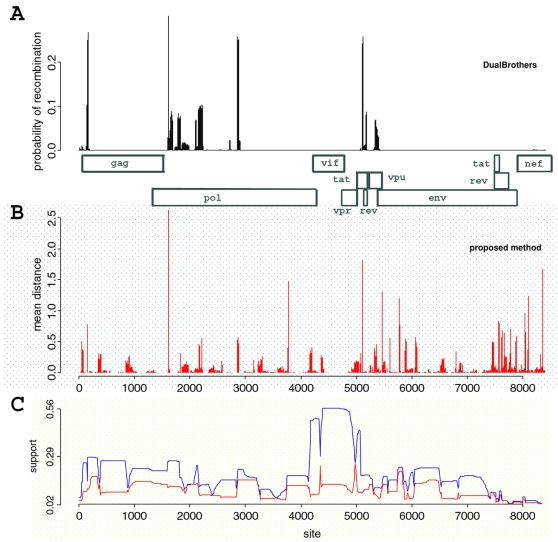
Evidence for recombination in HIV-1 genome sequences. Panel A shows the posterior probability of having a recombination breakpoint based on analyses using DualBrothers software, with a schematic representation of HIV-1 genes in scale. Since we analyzed each of the eight putative recombinant sequences independently in DualBrothers, this overall posterior probability is given by the sum of individual distributions. Panel B represents the posterior distribution of SPR distances between 10 bp segments as inferred by our method using samples from two independent runs. The horizontal axis is in the same scale as panel A. The support (posterior frequency) for the two most frequent topologies over segments is shown in panel C. For each segment we have the frequencies of the MAP topology (blue line) and the second most frequent topology (red line).

Figure 6 shows the support for recombination based on *d̂_SPR_* estimated by our method and the posterior probability of recombination estimated by DualBrothers program. The results indicate that regions with a higher probability of recombination (as indicated by DualBrothers, Figure 6A) were also detected by *d̂_SPR_* (Figure 6B). Therefore, both methods agreed in identifying recombination along HIV-1 sequences. Our method, however, detected much more phylogenetic heterogeneity that was undetected by the independent recombination analysis that we conducted with DualBrothers. This suggests that these recombinations do not involve parental references because, in our analysis with DualBrothers, we neglected the correlation between the recombinants, as is usually done when estimating the mosaic structure. Our result with the proposed Bayesian hierarchical method could indicate ongoing recombinations among CRF_12 viruses. The posterior distribution on the number of recombination breakpoints ranged between 30 and 47 with a mode (and median) of 37, while the sum of *d̂_SPR_* over the genome had a credibility interval of 55–77 SPR events with a mode of 65. This finding supports the existence of recombination hotspots since there are breakpoints harboring more than one recombination event. Examples include the beginning and the end of the *pol* gene and at the *tat*/*rev* genes (Figure 6B). The prior *μ*_0_ for the average branch length was around 0.025, and, since we have 11 sequences (19 branches), we have an expected substitution at every two sites, compatible with the values used in the simulations.

One difficulty in the analysis is to summarize the information from posterior topologies since we have a distribution of topologies for each segment. Our strategy was to observe the MAP topologies for each segment and to infer a possible recombination whenever neighboring MAP topologies disagree. The distances between MAP topologies for each segment indicate 49 and 52 breakpoints (for each independently sampled chain), an overestimation compared with sampled distances. This overestimation can be explained using Figure 6C, which shows the support (posterior frequency) for the two most frequent topologies for each segment along the alignment. There is virtually no difference in frequency between the MAP topology (most frequent) and some other topology after site 7500 of the alignment. This figure also shows which regions have a higher phylogenetic signal (for instance, between sites 4400 and 4900) and regions where the posterior distribution of trees is flatter and less reliable (such as the region around site 2000 or after 7500). A better strategy would be to summarize the distribution of topologies given the breakpoint pattern.

The accuracy of our recombination detection method is confirmed by observing the MAP topologies (Figure S2), that can be used to reconstruct the mosaic structure for a given choice of parentals. In fact, breakpoints detected by DualBrothers indicate inter-subtype recombinations according to our algorithm (observing the clustering of the recombinant sequences with the parental ones). For example, looking at the first two rows of figure S2, we observe that the clustering of the putative recombinants between the parentals C, B and F changes before sites 160, 1620, 1920, 2190 and 2870, in agreement with the breakpoints detected by DualBrothers. Even though *d̂_SPR_* is just an approximation to the recombination distance, it should not be inflated if there are no recombination hotspots. This can be explained by the fact that, when *d̂_SPR_* is larger than one, more than one recombination is necessary to explain the disagreement between the neighboring segments.

## Discussion

A recombination can be represented by an SPR move between rooted topologies; thus, the number of recombinations between neighboring sites can be estimated by the SPR distance between their underlying rooted trees [35], [36]. In this context, an explicit model for divergence times and evolutionary rates should be considered [37]–[39] since recombination can happen only between contemporary taxa. Neglecting branch lengths, SPR operations on rooted topologies always have an equivalent on unrooted topologies [40]-replacing the root node by one extant taxon in the unrooted case [41]. Then, the SPR distance between unrooted topologies that we approximate by *d̂_SPR_* can be regarded as a lower bound on the number of recombinations between sites, with the advantage that we do not need to disentangle times and rates. Our final target is not to find the recombination history, which can be better addressed by coalescent methods, but to estimate the most parsimonious number of recombinations necessary to explain the phylogenetic incongruence.

Our Bayesian hierarchical procedure not only detects the recombination breakpoints but also quantifies the disagreement between the trees. It therefore provides information regarding regions where recombinations occur frequently. The chance of correctly inferring the true tree is also higher than using other Bayesian procedures that neglect the similarity between trees on neighboring regions. Assuming a model of independent rates for each site and averaging over individual branch lengths as described in [42] proved to be useful in distinguishing recombination from non-random rate heterogeneity. It is worth mentioning that the integration *E_t_*[*Q*(*t*)] over individual branch lengths for a site (where *Q*(*t*) is the transition matrix as a function of branch length *t*) is not the same as assuming a fixed branch length *t* ^*^ for all branches since, in general, does not exist *t* ^*^ such that *E_t_*[*Q*(*t*)] = *Q*(*t* ^*^). In other words, marginalizing over branches is not equivalent to assuming the same branch length. Rather, it regards branches at each site as independent realizations from random variables. In simulations with 16 taxa, our procedure was robust to quantifying recombination (of which *d̂_SPR_* is a conservative measure) even when the real history is described by several nearby recombination breakpoints. These simulations also highlight how the SPR distance differentiates an ancestral recombination event (where *d̂_SPR_* = 1) from independent recombinations when we are confronted with several recombinant sequences sharing a similar mosaic structure. Distinguishing one ancestral recombination (shared among many sequences) from a recombination hotspot (many recombinations rising independently) can be difficult [22]. The robustness of our procedure comes from the fact that a breakpoint cannot be pinpointed with arbitrary precision, and the prior on the SPR distance accommodates this compromise. The amount of recombination over a region can, therefore, be quantified regardless of the number of breakpoints just by looking at the sum of *d̂_SPR_* over this region.

Credibility intervals can be constructed in the same way, by including all potential breakpoints (from larger to smaller posterior values), whose accumulated sum lies below some threshold. For example, the 95% credibility interval for *Y* breakpoints (where *Y* is the posterior mean of the total number of breakpoints) can be found by summing up the posterior frequencies of recombination for each segment, where these frequencies are given by the number of samples in which the segment had a distance larger than zero. If the sum is conducted for segments ordered from larger to smaller posterior frequencies, the credibility interval is composed by all segments such that the sum is smaller than 0.95 times *Y*. The same reasoning can be applied to the inference of recombination cold spots, regions where recombination might lead to disruption of protein function [43].

Applying our method to the HIV-1 dataset, we detected a number of recombination breakpoints much higher than the number detected when parental sequences are assumed. Consequently, there are many undergoing recombinations among BF viruses that may not involve the parental sequences. Moreover, since each SPR represents at least one recombination, the total number of approximate SPR moves is higher than the number of breakpoints (modal values of *d̂_SPR_* = 65 distributed among 37 locations). Thus, there are regions with recurrent recombinations in this dataset. One way of identifying these regions is to observe the segments in Figure 6B, where the mean *d̂_SPR_* is larger than one. Simply summing up the mean distances over a region provides an estimate of the minimum number of recombinations in that region. In the same figure (panel C), we can also have an idea about the most promising regions for further analysis. For instance, in the vicinity of *vif-vpr* genes, there is a region free from recombination that has one MAP topology with high support compared to other alternative trees. This region can, therefore, be used to compare the relatedness among distinct subtypes. In conclusion, our results show that HIV-1 variants with similar mosaic patterns may have been subjected to repeated events of recombination, which was not apparent from the mosaic structure.

## Methods

In the next subsection, we propose the approximate SPR distance between topologies. In the following two subsections, we describe the hierarchical Bayesian model, and, in the last two subsections, we describe the implementation of the sampling from the posterior distribution.

### SPR distance of topologies

#### Limits of existing distances in our framework

In an SPR operation, one edge of a topology together with one of the subtrees that it spans (the pruned subtree) is chosen, and this edge is then regrafted to a distinctly different edge of the remaining subtree [44]. The SPR distance *d_SPR_* is the minimum number of SPR operations needed to reconcile two trees on the same leaf set. For the general case, it can be calculated only by an exhaustive search [45], [46]. Heuristic approaches to calculate *d_SPR_* have been proposed in the context of horizontal gene transfer, where we can assume the topologies are rooted [25], [40], [47]–[50]. These procedures were designed for handling large topologies with a limited number or type of recombinations. The SPR distance is also related to the minimum number of recombination events that took place between two trees [51]. It is possible to use the SPR distance between unrooted topologies as the minimum number of recombinations [52], with the remark that the unrooted *d_SPR_* will always be a lower bound of the rooted *d_SPR_* since the rooting imposes a time constraint on events [41], [53]. There is a heuristic algorithm implementing the unrooted version of *d_SPR_*, but unfortunately with prohibitive time to be incorporated in our Bayesian analysis [54].

The most widely used topology metrics are the Robinson-Foulds distance and the maximum agreement subtree (MAST) distance. The Robinson-Foulds distance, or symmetric difference, is based on the split decompositions of the two topologies and is simply the number of edges that have no counterpart in the other topology [55]. Another measure is the MAST distance, which describes the number of leaves on the largest subtree that both topologies have in common [56]. Equivalently, the cMAST is the smallest number of leaves that should be removed from both topologies to make them agree. Unfortunately, neither of these distances is related to the SPR distance (see Figure S3).

Since we are interested in a conservative measure of recombination events, we adopt the *d_SPR_* between unrooted topologies. The actual number of recombinations will always be at least as large as our proposed *d̂_SPR_*. By doing so, our inference does not depend on resolving the root or on any assumption about potential parental sequences.

#### Proposed algorithm to approximate unrooted d_SPR_

**Figure 7:**
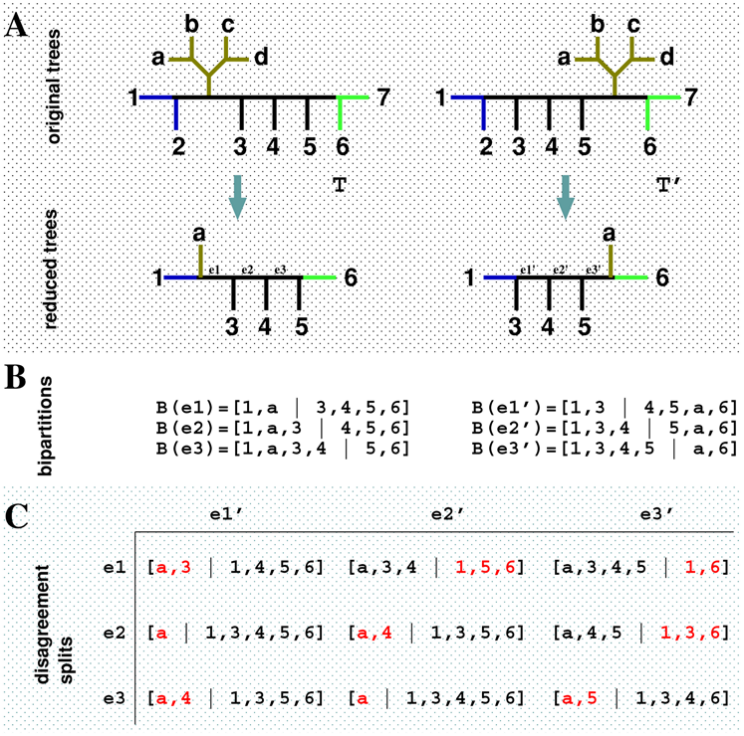
Example of one iteration of the algorithm that calculates *d̂_SPR_*. Panel A shows the topologies before and after the label compression in which the subtrees common to both topologies are replaced by a new leaf. Panel B shows the bipartitions induced by the edges of the compressed topologies, where the leaves (1), (a) and (6) represent the subtrees (1,2), ((a,b),(c,d)) and (6,7), respectively. Panel C represents the disagreement splits between all possible edge pairs in which the smallest leaf set for each disagreement split is shown in red. Ties, such as the disagreement between *e*_1_ and 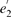, are broken by choosing the leaf set including some specific leaf, one in this case. We can observe that the smallest number of leaves causing a disagreement can be found by comparing *e*_2_ and 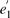 or, equivalently, *e*_3_ and 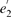. The associated subtree is the leaf (a), and, after its removal, both trees will be equivalent.

Here, we develop a novel algorithm that calculates the approximate SPR distance *d̂_SPR_* between unrooted topologies through a label compression technique in which equal subtrees in both topologies are replaced by a new leaf [45]. Recalling that a split, or bipartition, is a description of the leaves that become disconnected by removal of the edge it represents, an unrooted binary topology *T* on *N* leaves can be uniquely represented by its split set *B*(*T*) = {*B*(*e*_1_),…,*B*(*e_N_*_−3_)} if we consider only its internal edges *e*_1_,…,*e_N_*_−3_ [57]. Namely, a bipartition *B*(*e_i_*) defined by an edge *e_i_* can be represented by
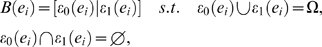
where *ε*_0_(*e_i_*) and *ε*_1_(*e_i_*) are the leaves separated by edge *e_i_*, and Ω represents the whole set of leaves such that |Ω| = *N*. For given two topologies *T* and *T*′, we can then classify its edges *B*(*T*) and *B*(*T*′) into equivalent *B_E_*(*T*), *B_E_*(*T*′) and nonequivalent edges *B_N_*(*T*), *B_N_*(*T*′). They represent the set of identical and distinct edges on both topologies as
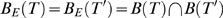
and
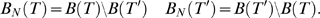
The number of nonequivalent edges between the topologies (|*B_N_*(*T*)|+|*B_N_*(*T*′)|) is their (unnormalized) Robinson-Foulds distance [55]. For binary trees, as is always the case in our study, we also have that |*B_N_*(*T*)| = |*B_N_*(*T*′)|. The label compression can then be accomplished by iteratively looking at the bipartitions in *B_E_*(*T*) where there is *ε*_0_(*e_i_*) or *ε*_1_(*e_i_*) with exactly two leaves and then replacing all occurrences of these leaves by a new leaf. Ties (when both |*ε*_0_(*e_i_*)| = 2 and |*ε*_1_(*e_i_*)| = 2 are broken by an arbitrary ordering of the leaves, and we acknowledge that this may be a poor solution. Figure 7A shows an example of such a label compression, where we can observe that the number of SPR events is not affected [45]. In the third top-down panel in this example, we show the bipartitions representing the (reduced) topologies.

For *B*(*e_i_*)∈*B_N_*(*T*) and 
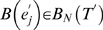
, let us define a disagreement split 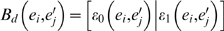, where
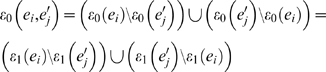
and
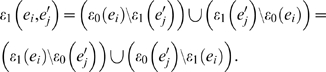

*B*(*e_i_*) and 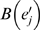 become the same when we consider only the complementary set of 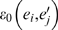 or 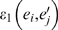. Either of them is the minimal set which satisfies this property. For example, in Figure 7, we have that the disagreement split between *e*_1_ and 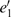 will have leaf sets
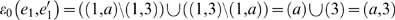
and
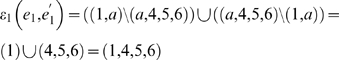
After calculating the disagreement split between all pairs of edges, we elect the smallest set of leaves found among them (that is, *ε*_0_(.,.) or *ε*_1_(.,.)), with ties broken by same leaf ordering as previously described. Figure 7C shows all disagreement splits in our example, where the smallest leaf set (actually just one leaf, after the tree reduction) is depicted in red. This elected set of leaves is then removed from both topologies, and *d̂_SPR_* is increased by one. This procedure of label compression and removal of the smallest leaf set in disagreement splits is repeated until all edges are in agreement between the topologies (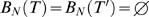). The approximate SPR distance between the topologies will then be the iteration count of the procedure.

Assuming that the comparison between edges can be done in constant time *O*(1), the procedure then has complexity *O*(*d_SPR_N* ^2^) for distinct trees. We have tried several other ad-hoc procedures, including MAST distance on reduced trees, but the one presented here was empirically the most successful. One simple case where our procedure fails is when the smallest set of leaves has two pruned subtrees. Our procedure counts it as one SPR, and taking the number of leaves into account decreases the performance for many other cases. The program can be extended to show the leaves that were actually removed as opposed to showing the distance, but we must bear in mind that there could be several SPR histories leading to the same minimum number.

### Prior distribution of the distances as a penalty against recombinations

We introduce a prior distribution for the SPR distance between the topologies of neighboring segments in the hierarchical Bayesian framework. This prior imposes a penalty against inconsistencies of topologies that require too many SPR operations to be resolved. Our assumption is that the number of SPR moves between unrooted topologies is related to the minimum number of recombinations. The strength of the penalty is expressed as a hyper-parameter. By introducing a hyper-prior, the value of the hyper-parameter is estimated as a posterior distribution.

Denoting the SPR distance *d̂_SPR_* at breakpoint *i* by *d_i_*, our prior distribution is described as a modified truncated Poisson:
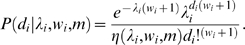
Here, 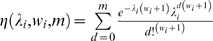. is the normalizing constant to account for the fact that any two topologies with *N* taxa cannot have an SPR distance larger than *m* = *N*−3, and *w_i_* is the weight on the penalty. Poisson distribution has the property of equality between the variance and mean, while the negative binomial distribution is often used to take account of over-dispersion compared with the Poisson distribution. In our case, we can make the segments arbitrarily short, even to 1 bp, as long as the computation is feasible. When the sequence is divided into short segments, it is reasonable to assume that the neighboring segments share the same topology in most cases. Even the Poisson distribution with a mean close to zero may not reflect this expectation sufficiently. The hyper-parameter *w_i_* of our modified Poisson distribution will easily adapt to the analysis of short segments since it induces an under-dispersed distribution compared to the Poisson. The prior on the total number of SPR events is not given explicitly since it is determined by this prior probability on the number of SPR moves per segment. The Bayesian hierarchical model incorporates further hyper-priors to account for the uncertainty on the strength of the penalty. That is, *λ_i_* and *w_i_* follow gamma distributions whose hyper-parameters *α_λ_*, *β_λ_*, *α_w_* and *β_w_* are shared across segments. This choice of hyper-priors, together with the “penalty” parameter *w_i_*, can take into account under- and over-dispersion of the SPR distance distribution compared to the Poisson distribution.

### Marginal likelihood and the prior for rate heterogeneity among sites and lineages

The whole alignment *X* is assumed to be decomposed into *K* consecutive segments. Neighboring segments may have different topologies due to recombinations. These segments can be arbitrarily small and should represent all regions with a potentially conflicting phylogenetic signal since our procedure estimates the recombinant regions as a subset of *K* and fixes evolutionary parameters within a segment. At the same time, since the speed of the algorithm is greatly affected by the number of segments, a reasonable choice for the number of segments should be made, with one segment per site in the ideal case. To achieve the robustness against rate heterogeneity, we assume that the evolutionary rate matrices are stochastically distributed among segments.

We use the standard evolutionary model, where the nucleotide substitution process at a given site is described by a continuous-time Markov chain and a phylogenetic tree describing the ancestral relations between extant taxa [58]. The evolutionary process of the segment *i* (*i* = 1,…,*K*) is assumed to follow the HKY model [59]. Each segment has its own ratio *κ_i_* (*i* = 1,…,*K*) of transitions to transversions, and the equilibrium frequencies of nucleotides are shared among all segments.

We write *X* = (*X*_1_,…,*X_K_*) where 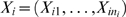 is the vector of alignment positions belonging to segment *i*. Denoting the topology of segment *i* by *T_i_* and the branch lengths of the *h*th alignment position of the segment by 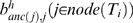, the conditional likelihood of segment *i* given the branch lengths is
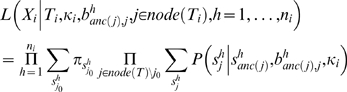
where *P* = (*s*_1_|*s*_0_, *b*, *κ_i_*) is the transition probability from nucleotide *s*_0_ to *s*_1_, *node*(*T_i_*) is the set of nodes of the topology *T_i_*, and *j*_0_ is the root. If the tree is an unrooted tree, as always in our case, *j*_0_ is any of the internal nodes and *anc*(*j*) is the parental node of node *j*. The summation is over the states at the internal nodes, and the states at the terminal nodes should fit to the observed data.

If we assume, such as in [42] that all branch lengths follow an exponential distribution with mean *μ_i_* and are independent among branches as well as among alignment positions, then we have the marginal likelihood:
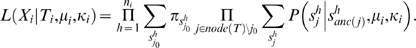
Here, *P*(*s*_1_|*s*_0_, *μ_i_*, *κ_i_*) is the marginal probability of transition from nucleotide *s*_0_ to *s*_1_, which can be calculated analytically through
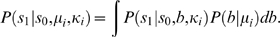
Since the marginalization is applied to each branch and to each site separately, the model allows the branch lengths to vary among sites while fixing the tree topology. When a large number of sequences are analyzed, our model assumes that the average branch length is common among sites within a segment but that it allows variable rates among segments. By partitioning the alignment into short segments (*e.g.*, less than 10 base pairs), our procedure takes account of rate heterogeneity among sites, with more accurate modeling for smaller segments. The marginalization over individual branches and the assumption of independence among segments should accommodate for rate heterogeneity among lineages and sites.

In our hierarchical setting, the transition∶transversion ratios *κ_i_* and the average substitution rates *μ_i_* are independent from each other and from the segments, and they follow exponential distributions with the means *μ*_0_ and *κ*_0_, respectively. Furthermore, *μ*_0_ and *κ*_0_ follow exponential distributions with the means 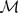 and 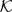, respectively. The equilibrium frequencies are calculated empirically from all segments.

### Sampling from the posterior distribution

If we represent the parameter vector by *θ*, then the posterior probability can be written as
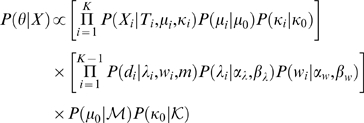
This distribution is numerically simulated by a Metropolis coupled Markov chain Monte Carlo (MC-MCMC) [60]. We employ a Metropolis-within-Gibbs sampler where all parameters are updated sequentially (systematic-scan) and the acceptance probability 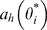 of a candidate state 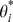 given its current state *θ_i_* is given by 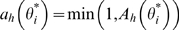 where
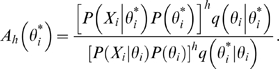

*P*(*θ_i_*) is shorthand for the prior distribution of parameter *θ_i_*, and *q*(·|*θ_i_*) is the proposal distribution. The parameter *h* (0<*h*≤1) is the heat value of the chain, and states sampled from the cold chain (*h* = 1) form an approximation of the posterior distribution. We run one cold and one heated (0<*h*_2_<1) chain concurrently, such that swap of states between them are accepted with the probability *a*(*h*_1_, *h*_2_) = min(1, *A*(*h*_1_, *h*_2_)), where
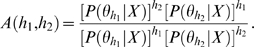
Here, 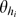 represents the parameter vector *θ* of chain *h_i_*.

For the continuous variables, namely *μ_i_*, *κ_i_*, *μ*_0_, *κ*_0_, *λ_i_* and *w_i_* a random variable u∼uniform (0,1) is drawn, and the candidate state is set as 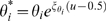, where 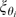 is a tuning parameter. The proposal ratio for these cases is 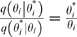.

We borrowed ideas from reversible-jump MCMC [42], [61], [62] to increase and decrease the number of recombination breakpoints and to change their location. Since updating all segments independently would have a very poor mixing, we always consider a block of consecutive segments that share the same topology. In our model, the number of parameters is constant, since even the topologies are distinct for every segment.

### Break-points update scheme

Let *j*_1_ and *j*_2_ be two segments such that *T_i_* = *T_i_*_+1_ for all *i*∈(*j*_1_,…,*j*_2_−1). If we call this topology *T_B_*, then our proposal topology 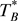 will be accepted with the probability
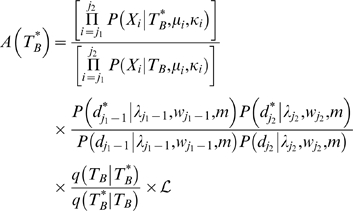
since all segments inside the block share the same topology. The constant 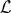 refers to the proposal ratio, which is usually one. If we have *T_i_* = *T_i_*_+1_ for all *i*∈(*k*_1_,…,*k*_2_−1) with both 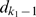 and 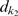 non-zero, then (*k*_1_,…,*k*_2_) is the largest non-recombinant region between *k*_1_ and *k*_2_. The removal of one recombination breakpoint is equivalent to choosing 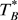 to be equal to 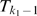 or 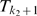 (with equal probability). The addition of a breakpoint can be attempted by setting *j*_1_ = *k*_1_+1,…,*k*_2_ or *j*_2_ = *k*_1_,…,*k*_2_−1 using the above formula, with 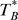 different from the border topologies 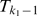 (if we chose *j*_1_) or 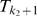 (if we pick up *j*_2_). If the proposal topology 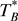 and the pertinent border are the same, it is equivalent to shifting the recombination breakpoint. If addition and removal of recombination breakpoints are attempted with equal probability, then detailed balance of the chain is satisfied. The exceptions are, thus, the regions before the first and after the last recombination breakpoints, where the frequency of removal updates is twice as large as the frequency of addition updates. For these cases, we set 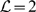 when proposing a breakpoint addition and 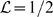 when proposing a deletion.

This breakpoints update scheme is performed in a symmetric scan (from the first breakpoint to the last and back). To decrease the autocorrelation between samples, we attempt to update all segments belonging to a non-recombinant region at every iteration by proposing a new topology 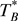. Here and at the breakpoint addition update, the new topology is chosen by applying one SPR move at the current topology *T_B_*. Another move is the nearest-neighbor interchange (NNI), a special case of SPR where the pruned subtree is neighbor to the regraft edge. The frequency *f_NNI_* at which an NNI move occurs can be set up to optimize the acceptance rate. Thus, when trying a breakpoint addition on a dataset with *N* taxa,
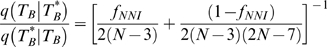
(1)since the numbers 2(*N*−3) and 2(*N*−3)(2*N*−7) correspond to the neighborhood sizes of the NNI and SPR moves, respectively [45] and the removal is deterministic. When attempting a breakpoint removal, the proposal ratio is the inverse of equation 1.

With this design, the proposal topology will always have *d_spr_* = 1 to the neighboring segment (since they differ by one SPR operation) when proposing a breakpoint addition. Alternatively, topologies with *d_spr_* = 1 may be rejected too often at recombination hotspot locations. To increase the acceptance rate in these cases, we developed a sampling procedure equivalent to the one proposed in [61]: after proposing a change in the number of breakpoints, we walk on the parameter space by a fixed number of steps. In practice, this means that several SPR or NNI moves are applied before the acceptance/rejection of the final state, decreasing the correlation between samples and allowing for neighboring segments to have a high SPR distance. Further details can be found in [61].

### Initial state sampled from heated “warm-up” chain

For both chains, their initial states are chosen independently based on cycles with variable temperature, whose initial values are picked up randomly from the priors or set to arbitrary values. When 0<*h*<1, as is usually the case in MC-MCMC, the updates are accepted more often. This allows for a better exploration of the parameter space. Conversely, using *h*>1 is more effective in finding a near-optimum state at the cost of low convergence if the chain is attracted by a local peak. In our simulated annealing scheme, the temperature *h_c_* at cycle *c* is given by *h_c_* = *h*_0_log(*c*+*C*) for some initial temperature *h*_0_>0 and *C*>0.

### Availability

The source code, datasets and scripts used in this study are available at http://corn.ab.a.u-tokyo.ac.jp/~leo/biomc2. The ANSI C source code is available under the GNU public license. In its current implementation a statistical software like R [63] is necessary to interpret the posterior distributions, but we are working on a way to circumvent this inconvenience.

## Supporting Information

Figure S1 Comparison between d_SPR_ and other distances for topologies with 64 taxa.
(0.30 MB PDF)

Figure S2 MAP topologies for the HIV-1 dataset, arbitrarily rooted at subtype C sequence.
(0.05 MB PDF)

Figure S3 Failure of distances in estimating the number of SPR operations.
(0.09 MB PDF)

Table S1 Description of the 11 HIV-1 sequences used in the recombination detection analysis.
(0.02 MB PDF)

## References

[1] Posada D (2002). Evaluation of methods for detecting recombination from DNA sequences: empirical data.. Mol Biol Evol.

[2] Posada D, Crandall KA, Holmes EC (2002). Recombination in evolutionary genomics.. Annu Rev Genet.

[3] Awadalla P (2003). The evolutionary genomics of pathogen recombination.. Nat Rev Genet.

[4] Fearnhead P, Harding RM, Schneider JA, Myers S, Donnelly P (2004). Application of coalescent methods to reveal fine-scale rate variation and recombination hotspots.. Genetics.

[5] Golding GB (1984). The sampling distribution of linkage disequilibrium.. Genetics.

[6] Griffiths RC (1981). Neutral two-locusmultiple allele models with recombination.. Theor Popul Biol.

[7] Hey J, Wakeley J (1997). A coalescent estimator of the population recombination rate.. Genetics.

[8] Hudson RR (1985). The sampling distribution of linkage disequilibrium under an infinite allele model without selection.. Genetics.

[9] Hudson RR (2001). Two-locus sampling distributions and their application.. Genetics.

[10] Kuhner MK, Yamato J, Felsenstein J (2000). Maximum likelihood estimation of recombination rates from population data.. Genetics.

[11] McVean G, Awadalla P, Fearnhead P (2002). A coalescent-based method for detecting and estimating recombination from gene sequences.. Genetics.

[12] Nielsen R (2000). Estimation of population parameters and recombination rates from single nucleotide polymorphisms.. Genetics.

[13] Wiuf C, Posada D (2003). A coalescent model of recombination hotspots.. Genetics.

[14] Siepel AC, Halperen AL, Macken C, Korber B (1995). A computer program designed to screen rapidly for HIV type 1 intersubtype recombinant sequences.. AIDS Res Hum Retroviruses.

[15] Salminen MO, Carr JK, Burke DS, McCutchan FE (1995). Identication of breakpoints in intergenotypic recombinants of HIV type 1 by bootscanning.. AIDS Res Hum Retroviruses.

[16] Weiller GF (1998). Phylogenetic profiles: a graphical method for detecting genetic recombinations in homologous sequences.. Mol Biol Evol.

[17] Grassly NC, Holmes EC (1997). A likelihood method for the detection of selection and recombination using nucleotide sequences.. Mol Biol Evol.

[18] Husmeier D, Wright F (2001). Probabilistic divergence measures for detecting interspecies recombination.. Bioinformatics.

[19] Husmeier D (2005). Discriminating between rate heterogeneity and interspecific recombination in DNA sequence alignments with phylogenetic factorial hidden Markov models.. Bioinformatics.

[20] Husmeier D, Wright F (2001). Detection of recombination in DNA multiple alignments with hidden Markov models.. J Comput Biol.

[21] Minin VN, Dorman KS, Fang F, Suchard MA (2005). Dual multiple change-point model leads to more accurate recombination detection.. Bioinformatics.

[22] Minin VN, Dorman KS, Fang F, Suchard MA (2007). Phylogenetic mapping of recombination hotspots in human immunodeficiency virus via spatially smoothed change-point processes.. Genetics.

[23] Sierra M, Thomson MM, Rios M, Casado G, Castro RO (2005). The analysis of near full-length genome sequences of human immunodeficiency virus type 1 BF intersubtype recombinant viruses from Chile, Venezuela and Spain reveals their relationship to diverse lineages of recombinant viruses related to CRF12_BF.. Infect Genet Evol.

[24] Hordijk W, Gascuel O (2005). Improving the efficiency of SPR moves in phylogenetic tree search methods based on maximum likelihood.. Bioinformatics.

[25] Nakhleh L, Ruths D, Wang LS (2005). RIATA-HGT: A fast and accurate heuristic for reconstructing horizontal gene transfer.. Computing and Combinatorics, Proceedings.

[26] Suchard MA (2005). Stochastic models for horizontal gene transfer: taking a random walk through tree space.. Genetics.

[27] Yang Z (1997). PAML: a program package for phylogenetic analysis by maximum likelihood.. Comput Appl Biosci.

[28] Fang F, Ding J, Minin VN, Suchard MA, Dorman KS (2007). cBrother: relaxing parental tree assumptions for Bayesian recombination detection.. Bioinformatics.

[29] Chan CX, Beiko RG, Ragan MA (2006). Detecting recombination in evolving nucleotide sequences.. BMC Bioinformatics.

[30] Rajaram ML, Minin VN, Suchard MA, Dorman KS (2007). Hot and Cold: Spatial Fluctuation in HIV-1 Recombination Rates.. Bioinformatics and Bioengineering, 2007 BIBE 2007 Proceedings of the 7th IEEE International Conference on.

[31] Huelsenbeck JP, Ronquist F (2001). MRBAYES: Bayesian inference of phylogenetic trees.. Bioinformatics.

[32] Ronquist F, Huelsenbeck JP (2003). MrBayes 3: Bayesian phylogenetic inference under mixed models.. Bioinformatics.

[33] Swofford DL (2002). PAUP*. Phylogenetic Analysis Using Parsimony (* and Other Methods) Version 4.0 b10.

[34] Gelman A, Rubin DB (1992). Inference from Iterative Simulation Using Multiple Sequences.. Statistical Science.

[35] Jotun Hein MHS (2005). Gene Genealogies, Variation and Evolution: A Primer in Coalescent Theory.

[36] Song YS, Hein J (2005). Constructing minimal ancestral recombination graphs.. J Comput Biol.

[37] Drummond AJ, Ho SY, Phillips MJ, Rambaut A (2006). Relaxed phylogenetics and dating with confidence.. PLoS Biol.

[38] Kishino H, Thorne JL, Bruno WJ (2001). Performance of a divergence time estimation method under a probabilistic model of rate evolution.. Mol Biol Evol.

[39] Thorne JL, Kishino H (2002). Divergence time and evolutionary rate estimation with multilocus data.. Syst Biol.

[40] Beiko RG, Hamilton N (2006). Phylogenetic identification of lateral genetic transfer events.. BMC Evol Biol.

[41] Song YS (2003). On the Combinatorics of Rooted Binary Phylogenetic Trees.. Annals of Combinatorics.

[42] Suchard MA, Weiss RE, Dorman KS, Sinsheimer JS (2003). Inferring spatial phylogenetic variation along nucleotide sequences: a multiple changepoint model.. J Am Stat Assoc.

[43] Lefeuvre P, Lett JM, Reynaud B, Martin DP (2007). Avoidance of protein fold disruption in natural virus recombinants.. PLoS Pathog.

[44] Felsenstein J (2004). Inferring phylogenies.

[45] Allen BL, Steel MA (2001). Subtree transfer operations and their induced metrics on evolutionary trees.. Annals of Combinatorics.

[46] Nakhleh L, Warnow T, Linder CR, St John K (2005). Reconstructing reticulate evolution in species-theory and practice.. J Comput Biol.

[47] Beiko RG, Harlow TJ, Ragan MA (2005). Highways of gene sharing in prokaryotes.. Proc Natl Acad Sci U S A.

[48] Ge F, Wang LS, Kim J (2005). The cobweb of life revealed by genome-scale estimates of horizontal gene transfer.. PLoS Biol.

[49] Hallett MT, Lagergren J (2001). Efficient algorithms for lateral gene transfer problems.. Proc Fifth Ann Intl Conf Comput Biol.

[50] MacLeod D, Charlebois RL, Doolittle F, Bapteste E (2005). Deduction of probable events of lateral gene transfer through comparison of phylogenetic trees by recursive consolidation and rearrangement.. BMC Evol Biol.

[51] Song YS, Hein J (2003). Parsimonious Reconstruction of Sequence Evolution and Haplotype Blocks: Finding the Minimum Number of Recombination Events..

[52] Hein J (1990). Reconstructing evolution of sequences subject to recombination using parsimony.. Math Biosci.

[53] Hein J (1993). A Heuristic Method to Reconstruct the History of Sequences Subject to Recombination.. Journal of Molecular Evolution.

[54] Hickey G, Dehne F, Rau-Chaplin A, Blouin C (2008). SPR Distance Computation for Unrooted Trees.. Evolutionary Bioinformatics 2008.

[55] Robinson DF, Foulds LR (1981). Comparison of phylogenetic trees.. Math Biosci.

[56] Steel M, Warnow T (1993). Kaikoura Tree Theorems-Computing the Maximum Agreement Subtree.. Information Processing Letters.

[57] Bryant D, Moulton V (2004). Neighbor-net: an agglomerative method for the construction of phylogenetic networks.. Mol Biol Evol.

[58] Felsenstein J (1981). Evolutionary trees from DNA sequences: a maximum likelihood approach.. J Mol Evol.

[59] Hasegawa M, Kishino H, Yano T (1985). Dating of the human-ape splitting by a molecular clock of mitochondrial DNA.. J Mol Evol.

[60] Altekar G, Dwarkadas S, Huelsenbeck JP, Ronquist F (2004). Parallel Metropolis coupled Markov chain Monte Carlo for Bayesian phylogenetic inference.. Bioinformatics.

[61] Al-Awadhi F, Hurn M, Jennison C (2004). Improving the acceptance rate of reversible jump MCMC proposals.. Statistics & Probability Letters.

[62] DiMatteo I, Genovese CR, Kass RE (2001). Bayesian curve-fitting with free-knot splines.. Biometrika.

[63] R Development Core Team (2008). R: A Language and Environment for Statistical Computing. Vienna: R Foundation for Statistical Computing. ISBN 3-900051-07-0.. http://www.R-project.org/.

