# Supplementary material for: Phylogenetic Detection of Recombination with a Bayesian Prior on the Distance between Trees
Source: PLoS One. 2008 Jul 9;3(7):e2651. doi: 10.1371/journal.pone.0002651 (PMC2440540; doi:10.1371/journal.pone.0002651)
Supplement: Figure S1 — Comparison between dSPR and other distances for topologies with 64 taxa. (0.30 MB PDF) [file pone.0002651.s001.pdf]

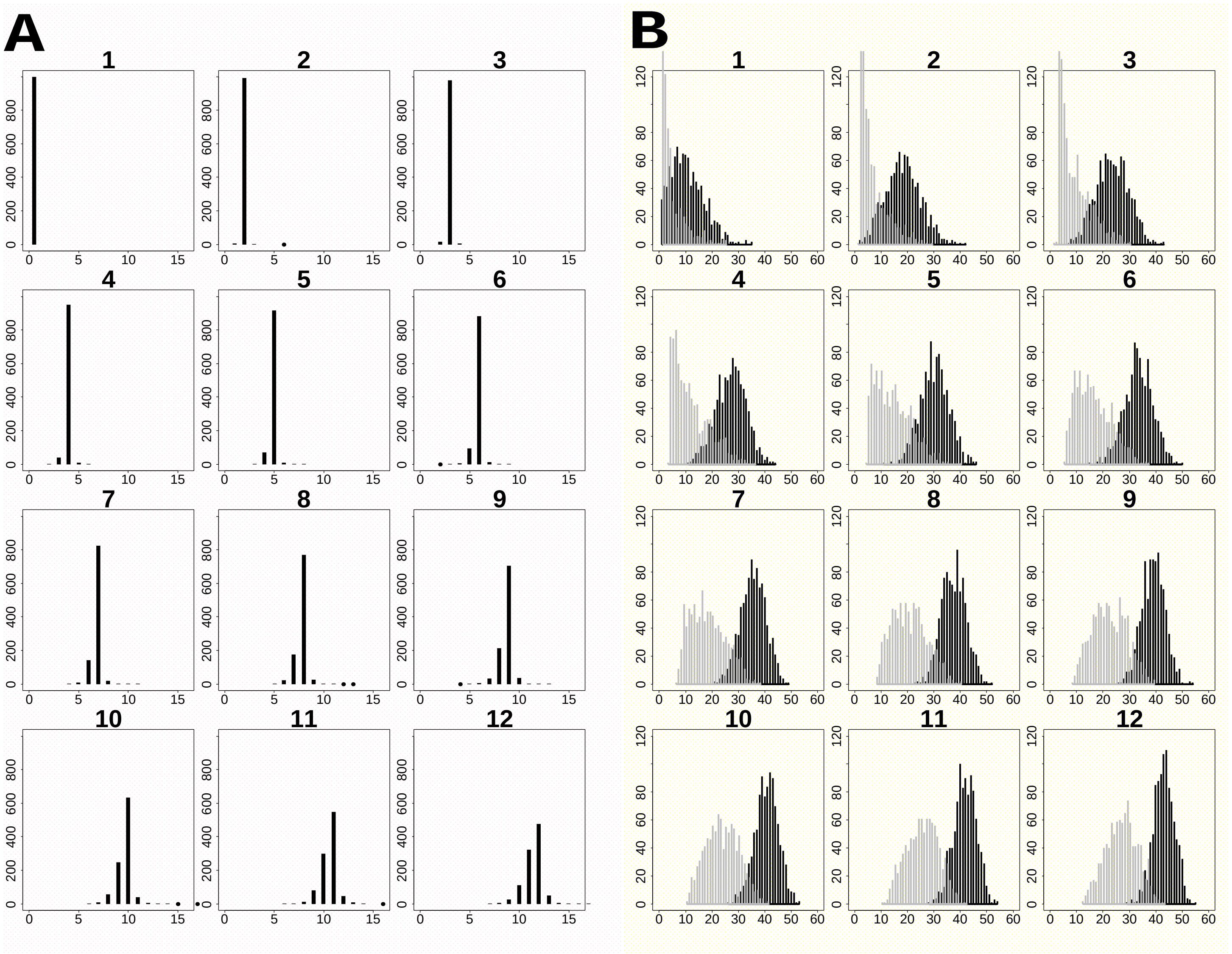

Figure S1
Comparison between $\hat{d}_{SPR}$ and other distances for topologies with 64 taxa. Each histogram represents 10^3 independent random topologies subject to SPR moves (number of applied SPR moves described on the top of each histogram). For each panel, the x-axis shows the estimated distances and the y-axis shows their frequencies over one thousand samples. In panel A we show our approximation to the SPR distance $\hat{d}_{SPR}$. Panel B shows the cMAST distance (in gray) and Robinson-Foulds distance (in black). The cMAST estimates were calculated with PAUP and the Robinson-Foulds distance can be computed as part of our procedure to estimate $\hat{d}_{SPR}$.
