# Supplementary material for: Phylogenetic Detection of Recombination with a Bayesian Prior on the Distance between Trees
Source: PLoS One. 2008 Jul 9;3(7):e2651. doi: 10.1371/journal.pone.0002651 (PMC2440540; doi:10.1371/journal.pone.0002651)
Supplement: Figure S2 — MAP topologies for the HIV-1 dataset, arbitrarily rooted at subtype C sequence. (0.05 MB PDF) [file pone.0002651.s002.pdf]

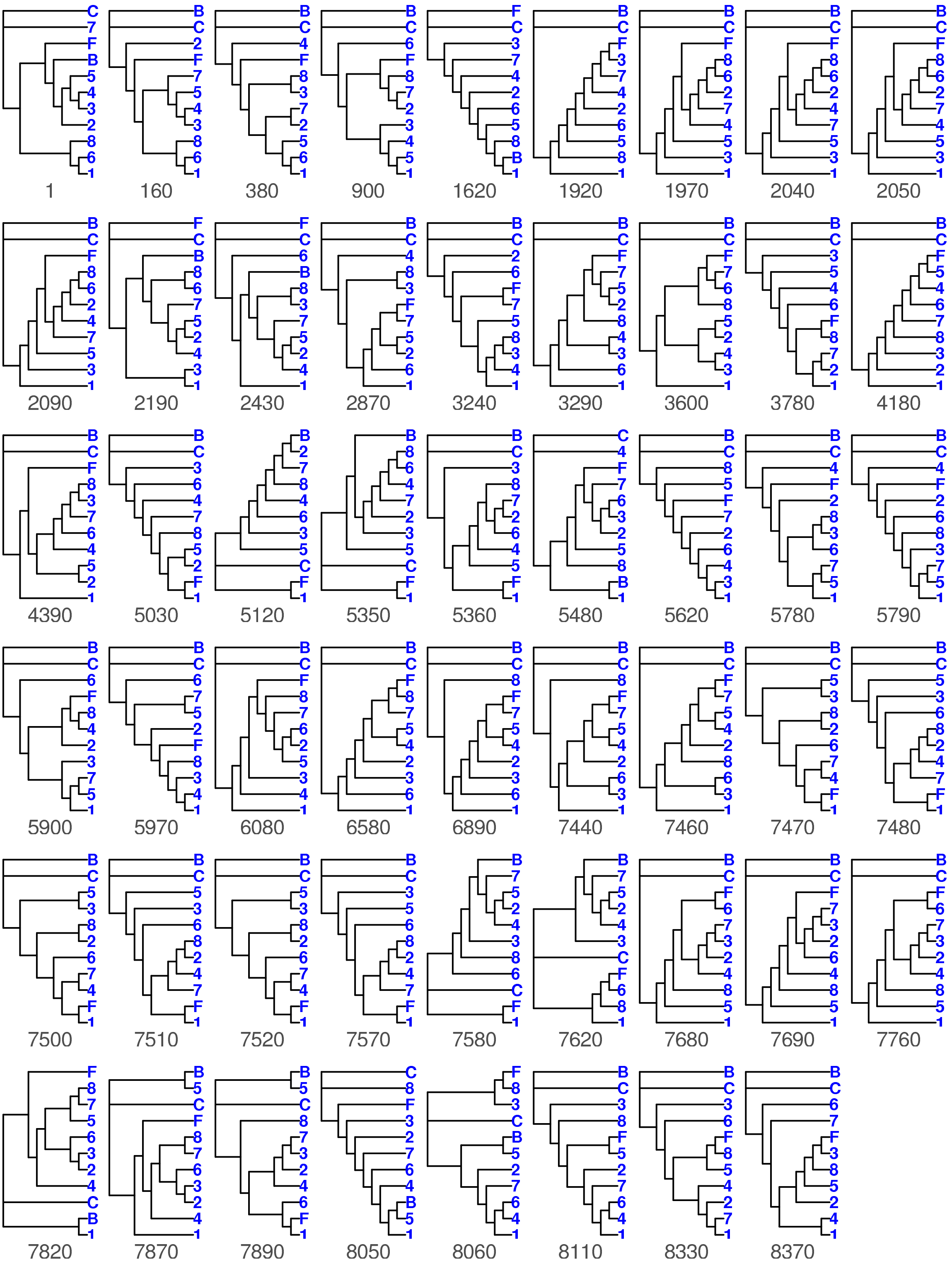

Figure S2

MAP topologies for HIV-1 dataset, arbitrarily rooted at subtype C sequence. Below each topology we have the first site of the region it comprises. The number at the tips correspond to the following sequences: (1) CH12, (2) 12_BF.AR.99.ARMA159, (3) 12_BF.UY.99.URTR35, (4) 12_BF.AR.97.A32879, (5) BF.AR.99.ARMA029, (6) BF.AR.99.ARMA097, (7) BF.AR.99.A027 and (8) BF.AR.99.A047 .
