# Supplementary material for: Phylogenetic Detection of Recombination with a Bayesian Prior on the Distance between Trees
Source: PLoS One. 2008 Jul 9;3(7):e2651. doi: 10.1371/journal.pone.0002651 (PMC2440540; doi:10.1371/journal.pone.0002651)
Supplement: Figure S3 — Failure of distances in estimating the number of SPR operations. (0.09 MB PDF) [file pone.0002651.s003.pdf]

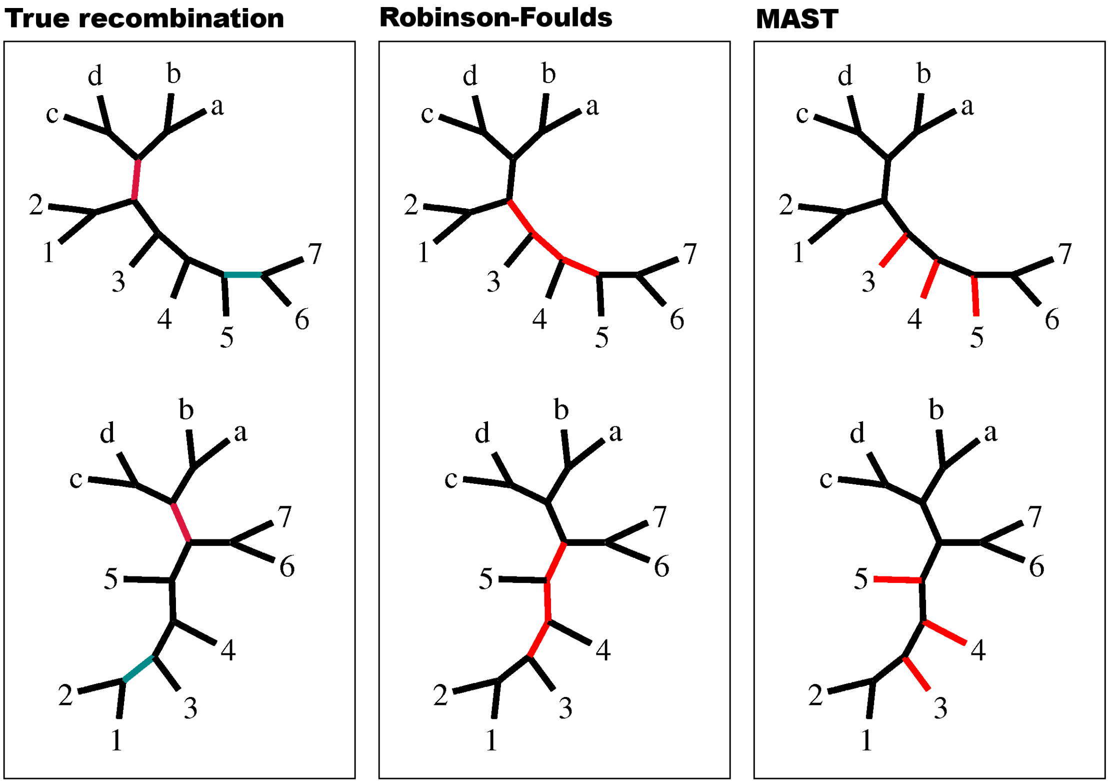


**Figure S3**
Failure of distances in estimating the number of SPR operations. The left panel is the true SPR event, with the pruned edge (red) and the regraft edges (blue). On the middle panel the edges in disagreement are in red, giving a Robinson-Foulds distance of three. The right panel shows the MAST in black, thus excluding three leaves (in red) from the agreement.
