# Supplementary material for: Phylogenetic Detection of Recombination with a Bayesian Prior on the Distance between Trees
Source: PLoS One. 2008 Jul 9;3(7):e2651. doi: 10.1371/journal.pone.0002651 (PMC2440540; doi:10.1371/journal.pone.0002651)
Supplement: Table S1 — Description of the 11 HIV-1 sequences used in the recombination detection analysis. (0.02 MB PDF) [file pone.0002651.s004.pdf]

Table S1

Description of the 11 HIV-1 sequences used in the recombination detection analysis. The "code" field refers to the coding used when plotting the topologies.

Code	Sequence name	Accession number	Year of sampling	Subtype	Country
1	CH12	AY536235	2001	CRF_12-like	Chile
2	12_BF.AR.99.ARMA159	AF385936	1999	CRF_12	Argentina
3	12_BF.UY.99.URTR35	AF385935	1999	CRF_12	Uruguay
4	12_BF.AR.97.A32879	AF408629	1997	CRF_12-like	Argentina
5	BF.AR.99.ARMA029	AY037283	1999	CRF_12-like	Argentina
6	BF.AR.99.ARMA097	AY037280	1999	CRF_12-like	Argentina
7	BF.AR.99.A027	AF332867	1999	CRF_12-like	Argentina
8	BF.AR.99.A047	AF408627	1999	CRF_12-like	Argentina
F	F1.BR.89.BZ126	AY173957	1989	F1	Brasil
B	B.BR.89.BZ167	AY173956	1989	B	Brasil
C	C.92.BR025	U52953	1992	C	Brasil
